# Perceptual Assessment and Acoustic Voice Analysis as Screening Tests for Vocal Fold Paresis After Thyroid or Parathyroid Surgery

**DOI:** 10.1007/s00268-020-05863-x

**Published:** 2020-11-28

**Authors:** Maria Heikkinen, Elina Penttilä, Mari Qvarnström, Kimmo Mäkinen, Heikki Löppönen, Jussi M. Kärkkäinen

**Affiliations:** 1 Department of Otorhinolaryngology – Head & Neck Surgery, Kuopio University Hospital, PL 100, 70029 Kuopio, Finland; 2 Institute of Clinical Medicine, University of Eastern, Kuopio, Finland; 3 Department of Phoniatrics, Kuopio University Hospital, Kuopio, Finland; 4 Heart Center, Kuopio University Hospital, Kuopio, Finland

## Abstract

**Background:**

The aim of this study was to evaluate the reliability of clinician-based perceptual assessment of voice and computerized acoustic voice analysis as screening tests for vocal fold paresis or paralysis (VFP) after thyroid and parathyroid surgery.

**Methods:**

This was a prospective study of 181 patients undergoing thyroid or parathyroid procedure with pre and postoperative laryngoscopic vocal fold inspection, perceptual voice assessment using grade, roughness, breathiness, asthenia, and strain (GRBAS) scale and acoustic voice analysis using the multi-dimensional voice program (MDVP). Patients were divided into 2 groups for comparison; those with new postoperative VFP and those without. Potential screening tools were evaluated using the receiving operating characteristic (ROC) analysis.

**Results:**

Fourteen (6.6%) patients had a new postoperative VFP. Postoperative GRBAS scores were significantly (*P* < 0.05) higher in patients with VFP compared to those without. However, there were no statistically significant differences in MDVP values between the groups. Postoperative GRBAS grade score (cut off > 0) had the best sensitivity, 93%, for predicting VFP, but the specificity was only 50%. Postoperative jitter (cut off > 1.60) in MDVP had a good specificity, 90%, but only 50% sensitivity. Combining all the GRBAS and MDVP variables with *P* < 0.05 in the ROC analysis yielded a test with 100% sensitivity and 55% specificity.

**Conclusions:**

Physician-based perceptual voice assessment has a high sensitivity for detecting postoperative VFP, but the specificity is poor. The risk of VFP is low in patients with completely normal voice at discharge. However, routine laryngoscopy after thyroid and parathyroid surgery is still the most reliable exam for VFP screening.

## Introduction

Vocal fold paresis or paralysis (VFP) caused by recurrent laryngeal nerve injury is a well-known complication following thyroid or parathyroid surgery with incidence rates ranging from 1.4% to 38% (1–4). In most institutions, the risk of postoperative VFP is roughly 5% [1, 3–10]. Postoperatively, VFP can be asymptomatic and go undetected unless routine laryngoscopy examinations are performed [11, 12]. The postoperative assessment of vocal fold function with laryngoscopy is time-consuming, requires special equipment and may cause discomfort to the patient. Still, it is important for the patient and for the surgeon to know if this complication has occurred. There are currently no good alternatives to laryngoscopy in detecting VFP after surgery.

Postoperative VFP may manifest with audible voice changes. These changes may be observed perceptually by a person or by a voice analyzing program. The perceptual voice quality assessment can be done using the grade, roughness, breathiness, asthenia, strain (GRBAS) scale [13]. For more objective assessment, acoustic voice analysis can be performed using the multi-dimensional voice program (MDVP) system which is currently the most commonly used and cited acoustic analysis software [14].

The primary aim of this study was to evaluate the reliability of clinician-based perceptual voice assessment (GRBAS) and computerized acoustic voice analysis (MDVP) in screening of new VFP immediately after thyroid and parathyroid surgery (before discharge). The secondary aim was to study the correlations between these two methods postoperatively in patients with or without VFP.

## Material and methods

**Fig. 1:**
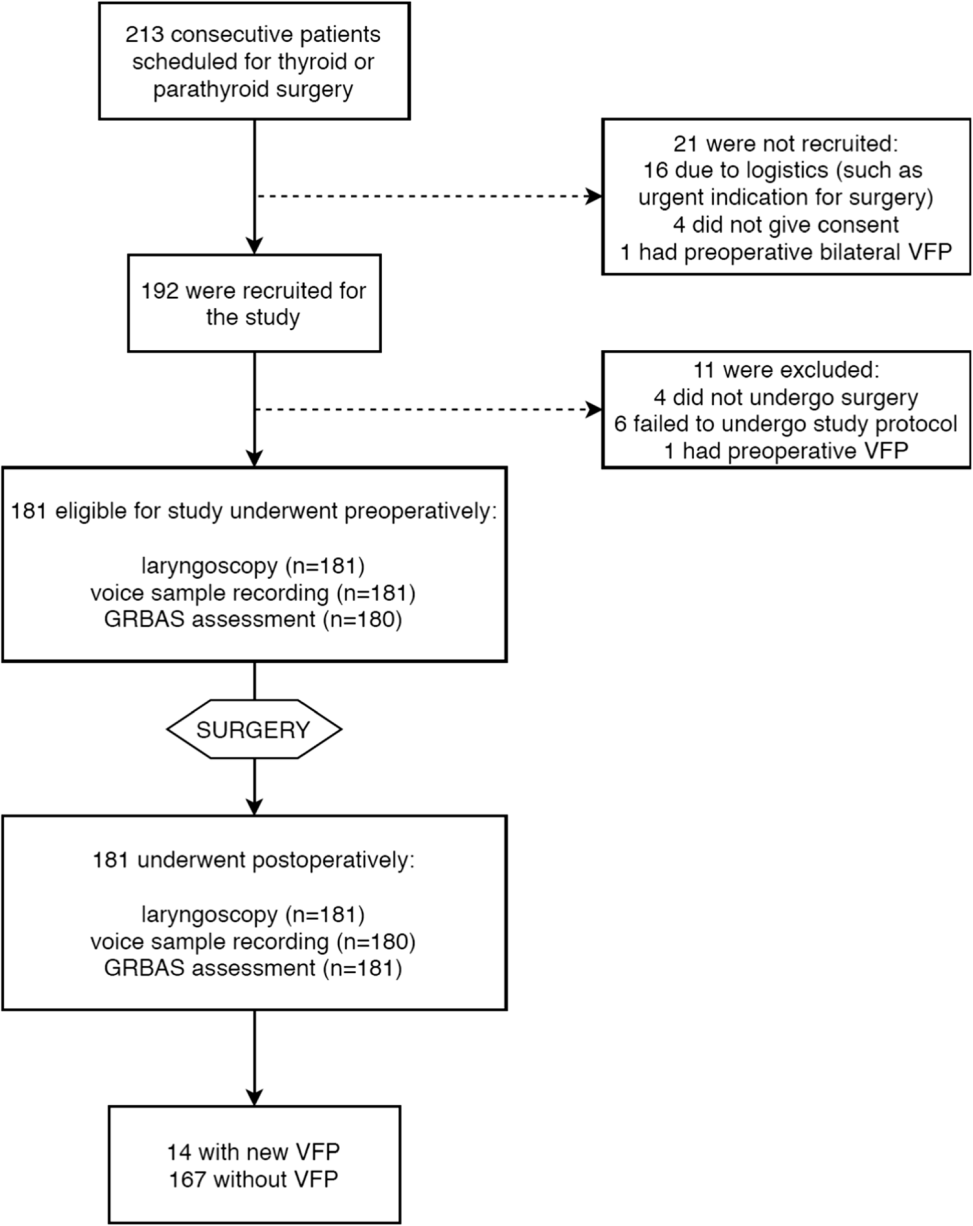
Flowchart of the study

This prospective study was approved by the institutional review board. Patients gave written informed consent. All consecutive patients undergoing thyroid or parathyroid surgery over a one-year study period in 2017 in a single academic hospital were considered for recruitment (*n* = 213). Twenty-one patients were ineligible for the study, and eleven patients were excluded after recruitment (Fig. 1). Finally, 181 patients were included in the study. The study patients underwent the voice quality assessments and vocal fold function examinations preoperatively and postoperatively 1.1 ± 0.3 days after surgery (prior to the discharge from the hospital). Preoperative laryngoscopy was performed to exclude preoperative VFP. Patients were divided into 2 groups based on postoperative lanrygoscopic examination; those with and those without a new postoperative VFP.

All patients underwent perceptual evaluation of voice by otolaryngologists using the GRBAS scale, before and after procedure. Nine of the examiners were in training and twelve were fully trained otolaryngologists. Otolaryngologists worked independent of the surgical team. Grade (*G*) is overall perceived degree of dysphonia, integrating all deviant components; roughness (R) is irregular fluctuation of the fundamental frequency; breathiness (*B*) is turbulence due to leakage of air through the insufficient glottic closure; asthenia (*A*) is weakness of voice, and strain (*S*) is perceived excess effort. Each parameter is scored using a scale of 0 to 3, where 0 is normal, 1 is slight disturbance, 2 is moderate disturbance, and 3 is severe disturbance. After the GRBAS voice rating, the otolaryngologist performed indirect laryngoscopy to evaluate vocal fold function. Fiberoptic laryngoscopy was used in 39 (22%) cases preoperatively and in 42 (23%) cases postoperatively when the visibility in indirect laryngoscopy was inadequate or suboptimal. New VFP was defined as immobility or insufficiency of the vocal fold.

Preoperative and postoperative voice recordings were performed on all patients by a trained nurse. Patients phonated a vowel, and 5 s samples were recorded with an iOS app called OperaVox (On PErson RApid VOice eXaminer, Oxford Research Wave Ltd, UK). The OperaVox app was installed on an iPad air 2 (Apple Inc., Cupertino, CA, USA). The device’s internal microphone as a recording system is compatible with a direct digitation method [15]. The iPad was placed in a tablet holder at 30 cm distance from the patient’s lips. Patients gave the voice samples in standing position unless their physical condition prevented it. OperaVox has a color bar indicator to measure instantaneous loudness of the voice, while the recording was performed. The recorded WAV files were exported to a MDVP workstation at a different location. The most high-quality 3 s of the recordings were acoustically analyzed with the MDVP software (KayPentax, NJ, USA). The analysis produces acoustic parameters including F0, jitter, shimmer, shimmer dB and noise to harmonic ratio (NHR). F0 is the mean frequency of mucosal vibrations of the vocal folds. Jitter and shimmer are perturbation measurements that measure cycle-to-cycle frequency and amplitude variation, respectively, in the analyzed voice sample. NHR is a measurement of the degree of hoarseness obtained by estimating the proportion of noise in the subject’s voice [16].

For validation of the described voice recording method, 20 randomly selected patients without VFP and 11 patients with VFP underwent a second round of postoperative voice sample recordings 2 weeks after surgery; this was done in a dedicated voice laboratory by a trained speech and language therapist. Patients gave a recording in an acoustically isolated booth. The voice sample was recorded using the iPad system and directly to the MDVP software using a condenser microphone. These recordings were then compared and studied for correlations of each of the recording parameters.

### Statistical analysis

All statistical analyses were performed using SPSS Statistics 24.0 (IBM Corp, Armonk, NY). Continuous variables were expressed as mean ± standard deviation (SD). The parameters were tested for normality by creating histograms. The group differences for normally distributed continuous variables were analyzed using the T-test. The correlation analysis was done by Pearson correlation coefficient test. Values between 0.1 and 0.3 were defined as mild, from 0.3 to 0.5 as moderate and more than 0.5 as strong correlation. Youden indexes and receiver operating characteristic (ROC) curves were generated to identify the critical values at which different variables were associated with VFP. The Youden index is an approach commonly employed to maximize both sensitivity and specificity and is calculated by summing the sensitivity and the specificity, and then subtracting number 1 from the result.

## Results

Altogether, 181 patients (mean age 58 ± 15 years, 87% female) were included in the study and underwent pre and postoperative examinations. The indications for surgery were goiter in 71 (39%), suspicion of malignancy in 39 (22%), malignant tumor in 6 (3%), hyperthyroidism in 25 (14%), and hyperparathyroidism in 40 (22%) patients. The type of the procedure was hemithyroidectomy in 86 (48%), total thyroidectomy in 51 (28%), isthmectomy in 4 (2%), and parathyroid procedure in 40 (22%) patients of which one was bilateral. The final pathological diagnosis was benign in 158 (87%) and malign in 23 (13%) patients. The mean length of hospital stay was 1.4 ± 1.5 days. Postoperatively, a new VFP was detected after 14 operations (10 paresis and 4 paralysis). The recurrent laryngeal nerve was inadvertently cut and noted in one patient. In the other patients with VFP, no injury of the RLN was recorded during the surgery.

**Fig. 2:**
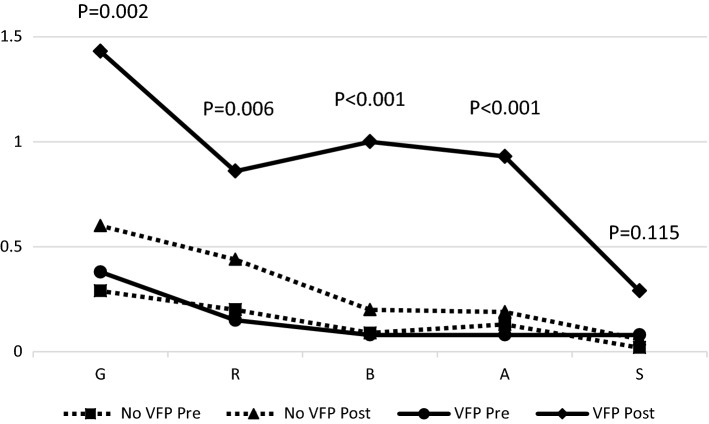
Pre and postoperative GRBAS scale scores (grade, roughness, breathiness, asthenia, strain) in patients with and without vocal fold paresis or paralysis (VFP). *P*-values represent the statistical significance of the change between pre and postoperative values in patients with VFP compared to those without

**Fig. 3:**
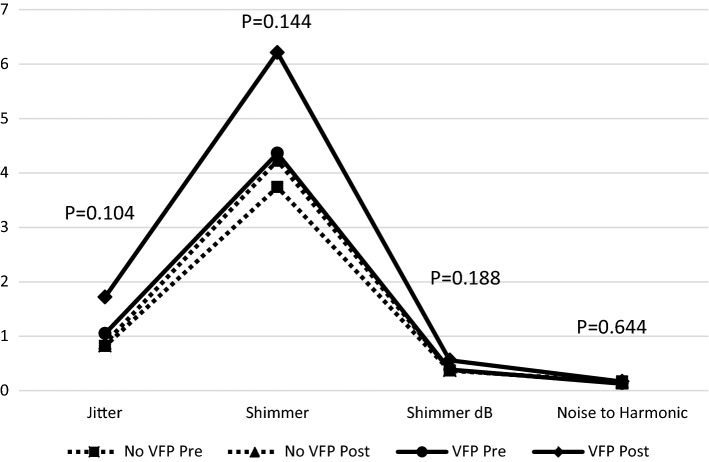
Pre and postoperative MDVP measures in patients with and without vocal fold paresis or paralysis (VFP). *P*-values represent the statistical significance of the change between pre and postoperative values in patients with VFP compared to those without

On perception analysis using the GRBAS scale, patients with VFP had significantly higher mean GRBAS scores postoperatively in all 5 domains compared to patients with no VFP (Table 1, Fig. 2). In addition, the mean change between the pre and postoperative GRBAS scores were greater among patients with new VFP; these differences were statistically significant in all except the mean change of strain (*S*). In the objective voice analysis using the recorded samples, no statistically significant differences were observed between patients with and those without VFP (Table 2, Fig. 3). However, there was a non-significant trend for more jitter in patients with VFP (*P* = 0.06).

**Table 1:** Mean preoperative and postoperative GRBAS scores for patients with vocal fold paresis or paralysis (VFP) and without (No VFP); the mean changes between the pre and postoperative values are shown in the right column

Scale item	Pre op	(Mean ± SD)		Post op	(Mean ± SD)		Voice change	(Mean ± SD)	
	No VFP	VFP	*P*-value	No VFP	VFP	*P*-value	No VFP	VFP	*P*-value
*G*	0.29 ± 0.49	0.38 ± 0.51	0.412	0.60 ± 0.69	1.43 ± 0.85	< 0.001*	0.31 ± 0.68	1.15 ± 1.07	0.002*
*R*	0.20 ± 0.43	0.15 ± 0.38	0.719	0.44 ± 0.66	0.86 ± 0.66	0.011*	0.23 ± 0.66	0.77 ± 0.73	0.006*
*B*	0.09 ± 0.29	0.08 ± 0.28	0.870	0.20 ± 0.44	1.00 ± 0.96	< 0.001*	0.11 ± 0.44	1.00 ± 1.08	< 0.001*
*A*	0.13 ± 0.74	0.08 ± 0.28	0.922	0.19 ± 0.42	0.93 ± 0.10	< 0.001*	0.05 ± 0.85	0.92 ± 1.12	< 0.001*
*S*	0.02 ± 0.22	0.08 ± 0.28	0.085	0.06 ± 0.24	0.29 ± 0.61	0.028*	0.04 ± 0.24	0.23 ± 0.73	0.115

*Statistically significant *P*-values

**Table 2:** Mean preoperative and postoperative F0, Jitter, Shimmer, Shimmer dB, and Noise to harmonic measurements for patients with vocal fold paresis or paralysis (VFP) and without (No VFP); the mean changes between the pre and postoperative values are shown in the right column

	Pre-op	(Mean ± SD)		Post-op	(Mean ± SD)		Voice change	(Mean ± SD)	
	No VFP	VFP	*P*-value	No VFP	VFP	*P*-value	No VFP	VFP	*P*-value
F0	233.48 ± 43.33	221.59 ± 61.56	0.490	216.98 ± 46.76	190.89 ± 58.38	0.051	− 16.47 ± 43.43	− 30.70 ± 29.06	0.231
Jitter	0.82 ± 0.96	1.05 ± 0.62	0.374	0.87 ± 1.14	1.72 ± 1.55	0.063	0.05 ± 1.37	0.67 ± 1.40	0.104
Shimmer	3.74 ± 1.70	4.36 ± 2.09	0.205	4.23 ± 3.24	6.21 ± 4.65	0.141	0.49 ± 3.31	1.85 ± 3.72	0.144
Shimmer dB	0.37 ± 0.33	0.39 ± 0.19	0.799	0.38 ± 0.29	0.56 ± 0.41	0.137	0.009 ± 0.43	0.16 ± 0.35	0.188
Noise to harmonic	0.17 ± 0.61	0.13 ± 0.03	0.795	0.14 ± 0.07	0.17 ± 0.08	0.109	− 0.04 ± 0.62	0.04 ± 0.07	0.644

GRBAS scores had mild to moderate correlation with all variables in the acoustic voice analysis except F0. The correlation was mostly moderate for* G* and* B*, and mild for* R*,* A*, and* S* (Table 3). Among patients without VFP, postoperative GRBAS scores had mild or moderate correlation with all voice analysis values except F0, whereas in patients with VFP, only few postoperative values had a statistically significant correlation due to the low number of patients in this group. However, in patients with VFP, grade (*G*), and breathiness (*B*) correlated strongly with jitter. The correlations between postoperative GRBAS and voice analyses are presented separately for patients with and without VFP in Table 4.

**Table 3:** Pearson’s correlation coefficients representing the correlation between the postoperative objective voice indices (F0, Jitter, Shimmer, Shimmer dB, and Noise to Harmonic) and postoperative GRBAS items for all patients

	*G*	*R*	*B*	*A*	*S*
F0	− 0.099	− 0.063	− 0.094	− 0.006	0.043
Jitter	0.343**	0.228**	0.452**	0.373**	0.245**
Shimmer	0.314**	0.203**	0.370**	0.296**	0.152*
Shimmer dB	0.317**	0.202**	0.369**	0.292**	0.141
Noise to harmonic	0.266**	0.173*	0.357**	0.241**	0.178*

*GRBAS*, Grade, Roughness, Breathiness, Asthenia, and Strain

*Correlation is significant at the 0.05 level (2-tailed)

**Correlation is significant at the 0.01 level (2-tailed)

**Table 4:** Pearson's correlation coefficients representing the correlation between the postoperative objective voice indices (F0, Jitter, Shimmer, Shimmer dB, and Noise to Harmonic) and postoperative GRBAS items for patients with vocal fold paresis or paralysis (VFP) and patients without (No VFP)

	*G*		*R*		*B*		*A*		*S*	
	No VFP	VFP	No VFP	VFP	No VFP	VFP	No VFP	VFP	No VFP	VFP
F0	− 0.082	0.147	− 0.109	0.662**	− 0.062	0.055	0.000	0.294	− 0.029	0.505
Jitter	0.248**	0.720**	0.222**	0.021	0.352**	0.728**	0.274**	0.590	0.225**	0.207
Shimmer	0.264**	0.418	0.237**	− 0.292	0.310**	0.482	0.257**	0.291	0.216**	− 0.179
Shimmer dB	0.267**	0.419	0.235**	− 0.289	0.309**	0.476	0.249**	0.302	0.202**	− 0.183
Noise to harmonic	0.230**	0.358	0.202**	− 0.380	0.326**	0.484	0.202**	0.321	0.229**	− 0.117

*GRBAS*, Grade, Roughness, Breathiness, Asthenia, and Strain

**Correlation is significant at the 0.01 level (2-tailed)

ROC analyses were performed in an attempt to discover a diagnostic tool for the screening of patients with VFP after surgery. Potential diagnostic tools and their evaluation methodology are presented in Table 5. Postoperative GRBAS grade score (cut off > 0) had the best sensitivity, 93%, but the specificity was only 50%. While postoperative jitter (cut off > 1.60) measurement had a good specificity (90%), the sensitivity was only 50%. The best Youden index was achieved in change of breathiness in GRBAS score (0.55). Combining 2 or more diagnostic tools did not yield a better Youden index.

**Table 5:** Assessment of potential diagnostic tools for vocal fold paresis or paralysis after thyroid or parathyroid surgery

	AUC (95% confidence intervals)	*P*-value	Cut off value	Sensitivity (%)	Specificity (%)	Number of missed VFPs (Out of 14)	Youden’s index
Post op GRBAS grade	0.763 (0.645, 0.882)	0.001	> 0	93	50	1	0.43
Post op GRBAS roughness	0.657 (0.533, 0.818)	0.029	> 0	71	65	4	0.36
Post op GRBAS breathiness	0.752 (0.594, 0.909)	0.002	> 0	64	82	5	0.46
Post op GRBAS asthenia	0.719 (0.552, 0.885)	0.007	> 0	57	82	6	0.39
Post op GRBAS strain	0.579 (0.408, 0.751)	0.325	> 0	21	94	11	0.15
Difference GRBAS grade	0.728 (0.575, 0.880)	0.006	> 0	69	66	4	0.36
Difference GRBAS roughness	0.696 (0.546, 0.845)	0.019	> 0	62	74	5	0.35
Difference GRBAS breathiness	0.768 (0.590, 0.946)	0.001	> 0	69	86	4	0.55
Difference GRBAS asthenia	0.732 (0.549, 0.915)	0.005	> 0	62	86	5	0.47
Difference GRBAS strain	0.559 (0.366, 0.751)	0.464	> 0	23	95	10	0.18
Postop F0	0.616 (0.439, 0.793)	0.150	< 162.05	50	89	7	0.39
Postop jitter	0.675 (0.500, 0,849)	0.030	> 1.60	50	90	7	0.40
Postop shimmer	0.623 (0.452, 0.795)	0.126	> 5.05	50	79	7	0.29
Postop Shimmer dB	0.621 (0.448, 0.793)	0.135	> 0.50	50	79	8	0.29
Postop harmonic to noise	0.667 (0.503, 0.831)	0.038	> 0.15	50	84	8	0.34
Difference F0	0.624 (0.495, 0.754)	0.123	< -32.43	57	70	6	0.27
Difference jitter	0.617 (0.441, 0.792)	0.147	> 0.86	43	90	8	0.33
Difference shimmer	0.624 (0.453, 0.795)	0.124	> 2.11	50	82	7	0.32
Difference shimmer dB	0.626 (0.456, 0.796)	0.119	> 0.17	50	81	7	0.31
Difference harmonic to noise	0.704 (0.553, 0.854)	0.012	> 0.02	71	75	9	0.46

AUC area under the curve, VFP vocal fold paresis or paralysis

The validation of the recording technique showed strong correlation between all parameters recorded with iPad compared to those recorded directly to the MDVP software. The Pearson correlation coefficient was 0.95 for F0, 0.85 for jitter, 0.77 for shimmer, 0.84 for shimmer dB, and 0.75 for NHR.

## Discussion

This study demonstrated that the clinician’s perceptual assessment of the patient’s voice after thyroid or parathyroid surgery is sensitive in detecting most postoperative VFPs. If the GRBAS grade score (a composite of *R*, *B*, *A* and *S*) was more than zero, meaning that there was any significant disturbance in the patient’s postoperative voice, the sensitivity of this test being able to detect VFP was 93%. This means that 1 of the 14 postoperative VFP complications would have been missed without routine laryngoscopic examinations. However, the specificity of this test was only 50%. Therefore, using perceptual voice assessment as a screening tool, half of the patients would still have to undergo laryngoscopic examination after surgery. The additional value of objective voice analysis using MDVP parameters was minimal considering that the computerized voice analysis is more cumbersome to perform than the clinician-based assessment.

In addition to the present study, a few previous studies have demonstrated increased GRBAS scores in patients with VFP. Jesus and colleagues compared 17 patients with unilateral VFP with 43 controls; all GRBAS parameters were statistically significantly higher in the VFP group [17]. Furthermore, Jedra and colleagues examined 25 patients with iatrogenic VFP one to two days after the onset of speech impairment; all study patients had GRBAS grade score more than zero [18].

Iatrogenic VFP should be diagnosed early, preferably before discharge for 2 main reasons [19]. First, the surgeon gets immediate feedback which can help avoiding recurrent laryngeal nerve injuries in the future. Second, the patient has direct benefits from early diagnosis; aspiration problems may be prevented, symptomatic patients get referred to voice therapy early in the process, and surgical treatment will be considered in timely fashion, when needed [20, 21]. Even patients with asymptomatic VFP should be detected ahead of time. Initially, VFP may be asymptomatic because of compensatory movement of the contralateral vocal fold. However, symptoms may arise with aging when the compensation mechanisms become weaker. VFP that is detected at a later time may cause unnecessary etiological examinations.

While a patient with VFP may be asymptomatic, a patient with postoperative voice disorder may not necessarily have VFP [22]. Therefore, it is challenging to create a screening test for postoperative VFP which would be both sensitive and specific. In a previous prospective study by Ortega and colleagues including 64 patients undergoing thyroid surgery with pre and postoperative computerized acoustic voice analysis (a program created on the base of MDVP) and subjective GRBAS evaluation, the authors suggested that patients with normal findings in these tests may not need laryngoscopy to exclude VFP [23]. The sensitivity and specificity of GRBAS were 100% and 61% 1 week after the procedure and 45% and 98% for computerized acoustic voice analysis, respectively. The authors also noted that the sensitivity of computerized analysis might increase in a repeated examination 1 month after procedure. However, the study included only 5 patients with postoperative VFP, and therefore, the results should be interpreted with caution. On the other hand, our study showed similar results in the early postoperative period for GRBAS and MDVP as Ortega’s study. Performing these tests before discharge would be beneficial since the patients does not need to come back for the examination.

Combining 2 measures (presented in Table 5), 1 with good sensitivity and 1 with good specificity, such as GRBAS Grade and GRBAS Strain, did not give any better tool to screen VFP. However, a sum variable combining eleven independent variables achieved 100% sensitivity. Nevertheless, given that the specificity was only 55% and the calculation of the sum variable is fairly complex, this may not be a very practical tool for clinical use. Correlations between postoperative GRBAS scores and acoustic parameters differ between patients with and without VFP (Table 4). Patients with no VFP had correlation between nearly all GRBAS scores and acoustic parameters. In contrast, only 3 pair of GRBAS scores and acoustic parameters had correlation among patients with VFP. A possible explanation for the lower correlation of perceptual and computerized voice assessment in patients with VFP may be the difficulty to do an accurate objective analysis of a pathologic voice. Another reason could be that the number of patients with VFP was too small to show statistically significant correlation.

The low specificity of voice assessment may be explained by the high prevalence of voice disorders in the population. Nearly 8% of adults are experiencing voice problems including those who have no pathological findings in the larynx [24]. Furthermore, iatrogenic causes other than recurrent laryngeal nerve damage may cause voice changes after thyroid or parathyroid surgery, such as larynx irritation or trauma attributed to the endotracheal intubation. Intubation may cause a hematoma, laceration of vocal fold mucosa or muscle, and even subluxation of the arytenoid cartilage [25, 26]. This type of trauma may heal spontaneously. However, shortly after surgery it may cause voice changes which could be detected in MDVP voice analysis. Moreover, external branch of the superior laryngeal nerve may be damaged during the surgery. This damage is linked to cricothyroid muscle motility impairment, an altered frequency of voice, modified timbre, and deterioration in voice performance (high-pitched sounds) [19]. In addition, division, intraoperative fixation or injury of prelaryngeal strap muscles (sternohyoid, sternothyroid) leading to postoperative voice impairment has been described, and edema of the neural structures innervating the muscles needed for phonation may also cause voice changes [27]. Hence, causes other than VFP may induce changes in MDVP measures after surgery. Maeda and colleagues reported statistically significant worsening in parameters of acoustic voice analysis after thyroidectomy among 110 patients with no VFP suggesting that thyroidectomy has a distinct impact on voice quality even without recurrent laryngeal nerve injury [28]. In our study, we did not notice any significant changes in MDVP measures in patients without VFP after surgery.

### Study limitations

In this study, GRBAS scale was scored by 21 different otolaryngologist who were introduced to the use of the scoring system, but had little or no previous experience in GRBAS. In addition, the pre and postoperative assessments were not always conducted by the same physician. These factors may cause variability in GRBAS grading of the study patients. However, the clinician based GRBAS rating system has been associated high interobserver reliability in previous studies [29]. Indirect laryngoscopy was performed as the primary investigation to distinguish patients with or without VFP. Fiberoptic laryngoscopes were used in case of poor visibility in indirect laryngoscopy. In addition, we recognize a potential for bias as the same otolaryngologist performed GRBAS assessment and the subsequent laryngoscopy. If the patient has no voice abnormality, the investigator could be tempted to skip the time-consuming fiberoptic laryngoscopy in case of suboptimal visibility in the indirect laryngoscopy. However, we think that the risk of this bias is low in our study because the otolaryngologists in our institution have performed routine vocal fold examinations pre and postoperatively for 200 annual patients undergoing thyroid and parathyroid surgery for several years before the study. Finally, the low number of patients with VFP event in this study may underestimate the value of the screening tests because of the possibility of type 2 statistical error.

## Conclusion

Perceptual voice assessment with or without objective acoustic analysis has a high sensitivity for detecting postoperative VFP, but the specificity is poor. It is possible that using perceptual voice assessment, half of the routine laryngoscopic examinations could be avoided after thyroid and parathyroid surgery if laryngoscopy was omitted in patients with completely normal voice at discharge; the risk of postoperative VFP in these patients is low, but not zero. The utility of computerized acoustic voice analysis alone was limited. Further studies are needed to create an accurate screening test for postoperative VFP. Meanwhile, routine laryngoscopy after thyroid and parathyroid surgery is still the most accurate test for VFP screening.
